# Supplementary figures and images for: Highly specific and sensitive detection of Burkholderia pseudomallei genomic DNA by CRISPR-Cas12a
Source: PLoS Negl Trop Dis. 2022 Aug 29;16(8):e0010659. doi: 10.1371/journal.pntd.0010659 (PMC9423629; doi:10.1371/journal.pntd.0010659)

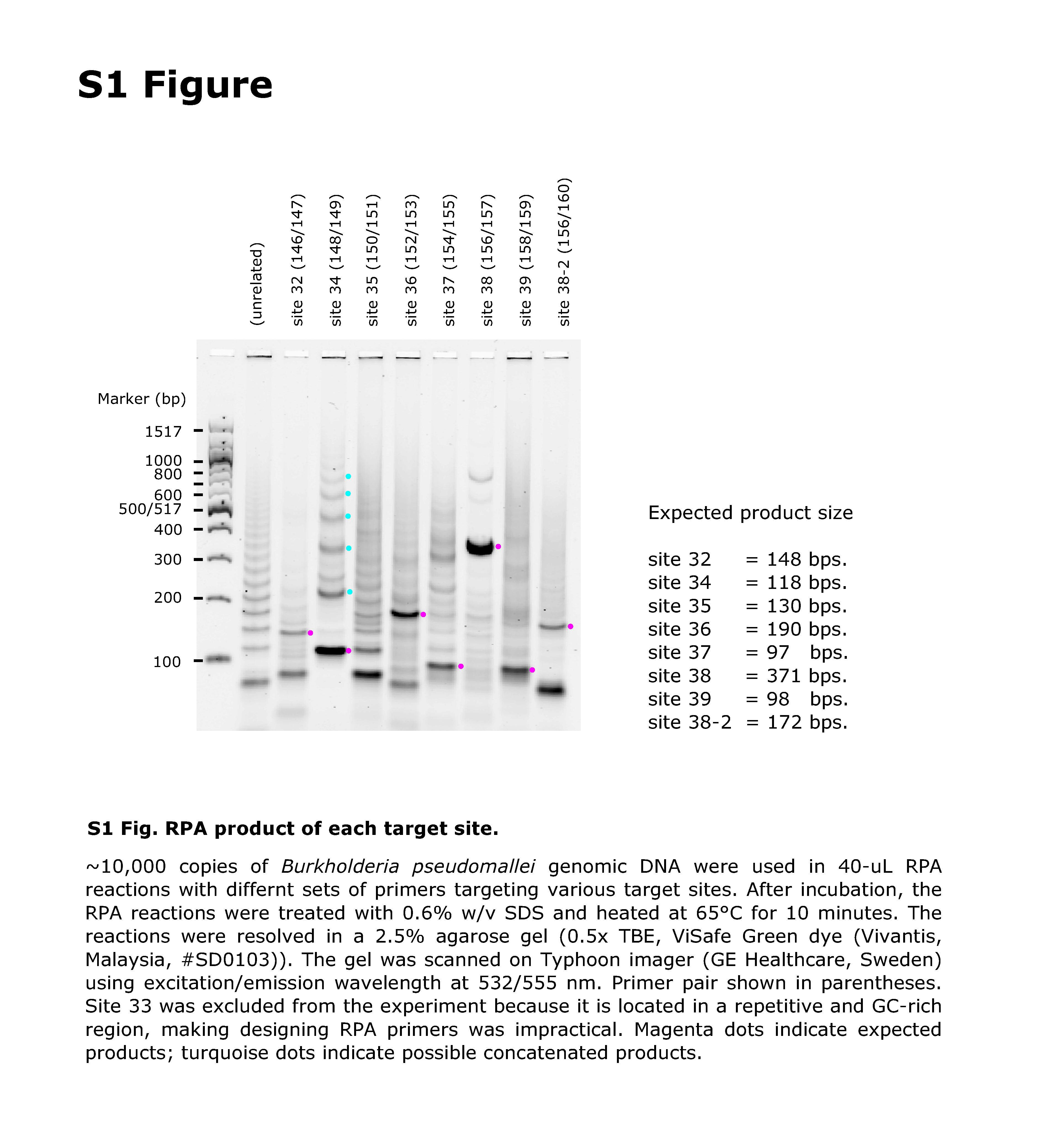

Supplement: S1 Fig — (TIF) [file pntd.0010659.s001.tif]

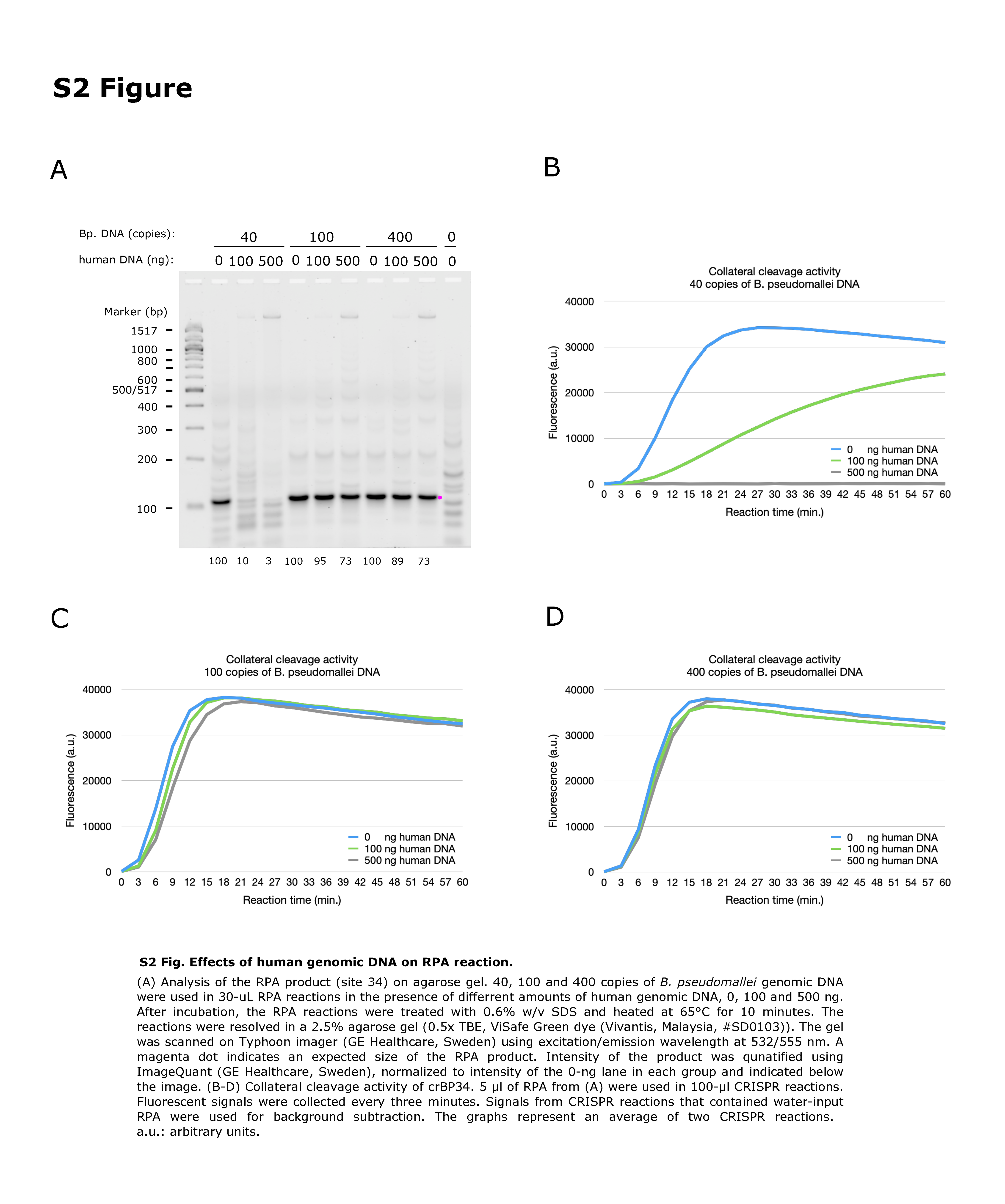

Supplement: S2 Fig — (TIF) [file pntd.0010659.s002.tif]

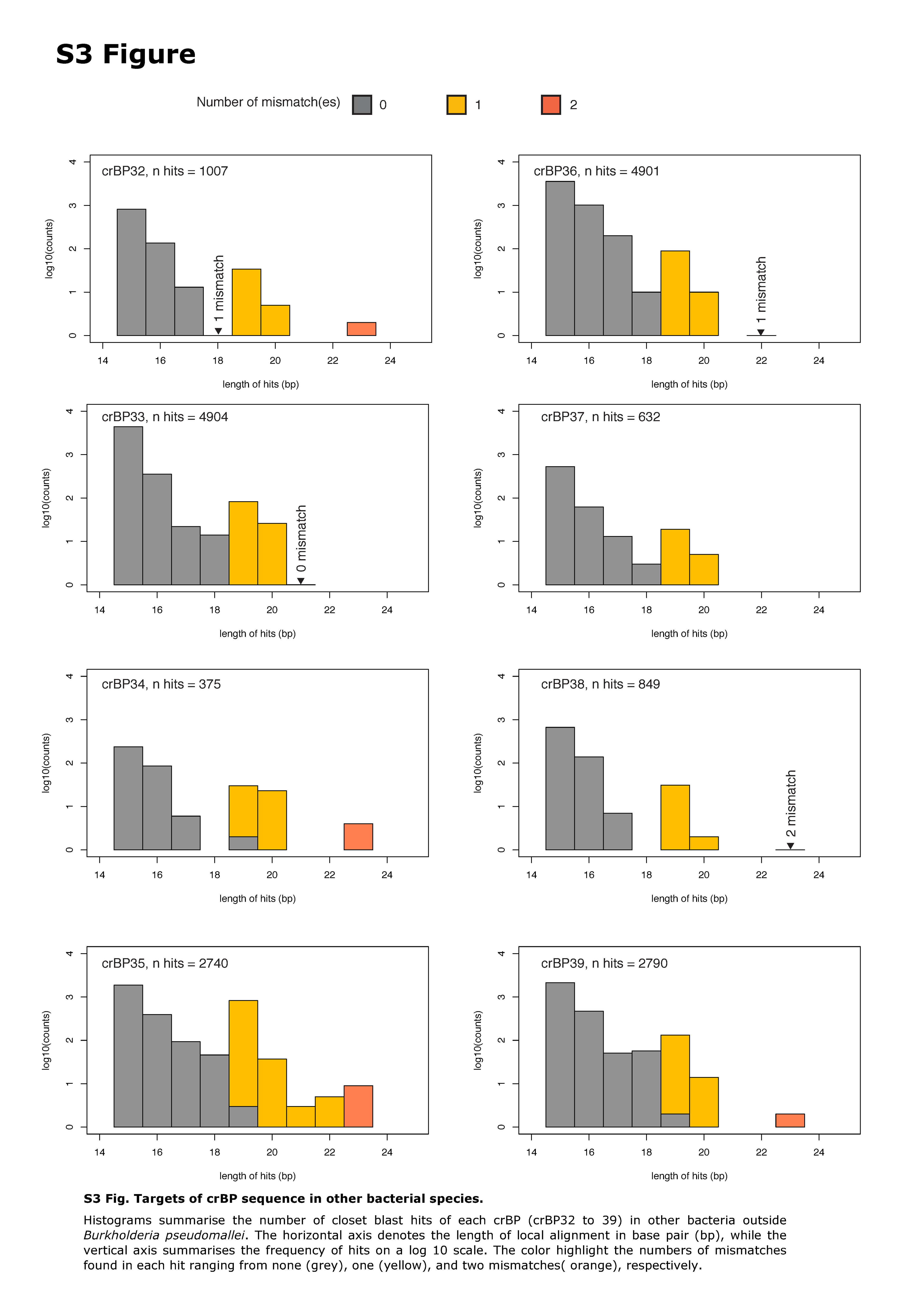

Supplement: S3 Fig — (TIF) [file pntd.0010659.s003.tif]

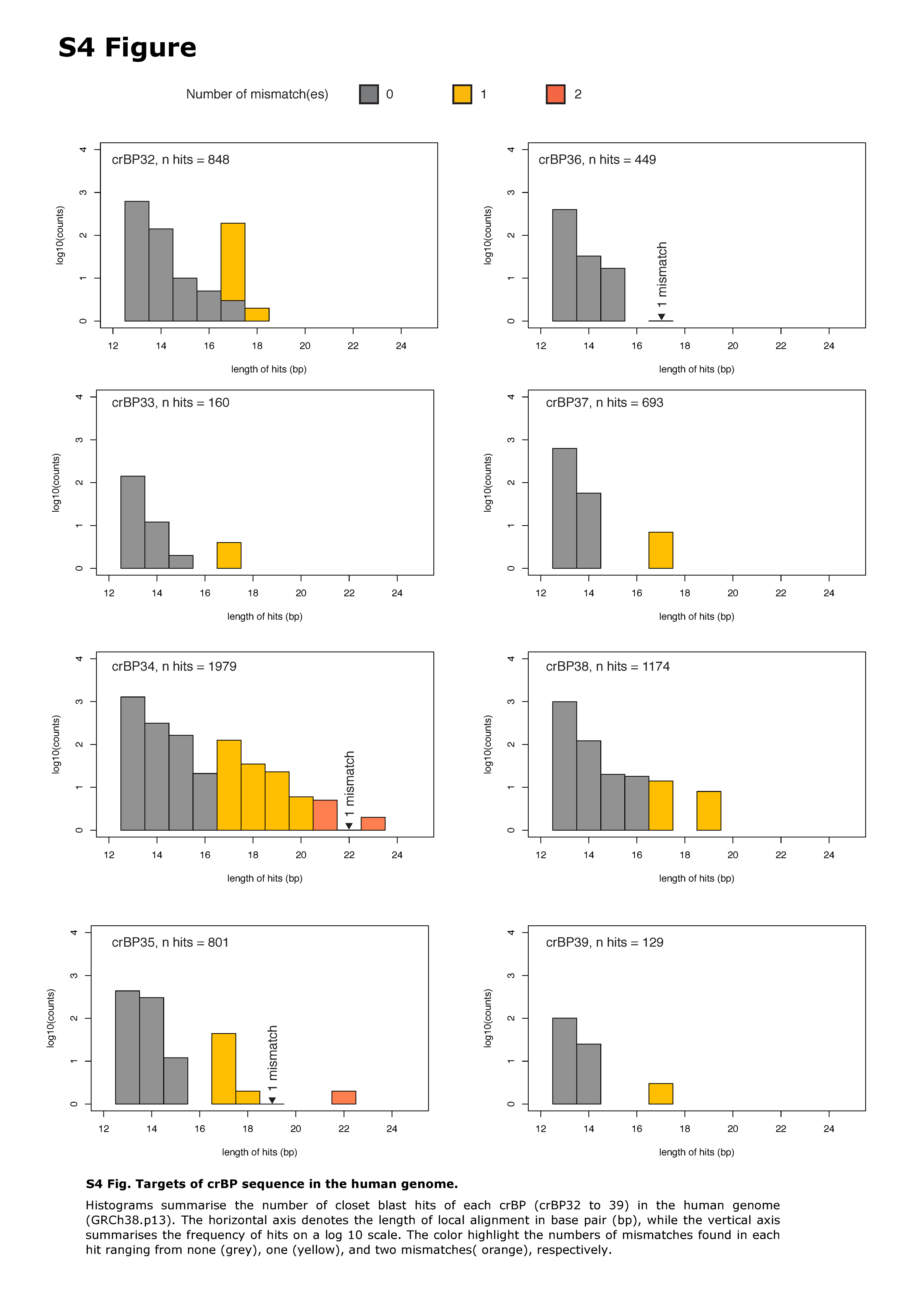

Supplement: S4 Fig — (TIF) [file pntd.0010659.s004.tif]
