# Supplementary material for: Highly specific and sensitive detection of Burkholderia pseudomallei genomic DNA by CRISPR-Cas12a
Source: PLoS Negl Trop Dis. 2022 Aug 29;16(8):e0010659. doi: 10.1371/journal.pntd.0010659 (PMC9423629; doi:10.1371/journal.pntd.0010659)
Supplement: Attachment — Submitted filename: Response to reviewers comments.pdf [file pntd.0010659.s010.pdf]

Responses to reviewers' comments

Please note:
1) The reviewers' original comments are in **black**.
2) The authors' responses are in **green**.
3) Line numbers in the authors' responses are referred to the "clean" version of the manuscript (we observed shifts in line numbers after accepting track changes).

****

METHOD

Reviewer #1: The manuscript entitled with " Highly Specific and Sensitive Detection of Burkholderia pseudomallei Genomic DNA by CRISPR-Cas12a" submitted by Wongpalee et.al., presents a novel method that circumvents long time amplification issues by utilizing CRISPR-Cas12a coupled with isothermal amplification of a target DNA. Although the article has scientific rigor, few flaws need to be corrected before it can be considered for publication.

• Aspects of CRISPR technology for diagnostics and treatment needs more advanced elaboration.
https://link.springer.com/article/10.1007/s40097-022-00472-7
We appreciate the reviewer for the comment. We have re-written the paragraph concerning CRISPR-Cas (Line 100-127) so that it incorporates the reviewer's suggestion and appropriate citations.

• Define the "frequency" and the time duration for sonication in the method protocol.
We apologize for being unclear. We have added sonication parameters in the Materials and Methods section.

• Report the length, melting temperature and probes of the primers used in the RPA and the region used for the amplification.
We have provided the information of primers and probes in a supplementary table (Table S3)

• Correct the "μl" spelling throughout the document
We thank the reviewer for pointing this out. We have corrected the spelling throughout the document.

• Define the importance of the target sites "crBP34", "crBP36", "crBP38" and its role as a controlling factor for melioidosis.
We thank the reviewer for helping us make this manuscript clearer. All eight candidates are located in both coding and non-coding regions of the genome. Unfortunately, functions of many of these loci in melioidosis are not well characterized. Nonetheless,

we have edited Fig 1D to include genomic location, annotation and product description of encoded protein (if any) for all final candidates.

• Are the researchers making any modifications in the purification protocol, as there is a lack of component "sodium phosphate" in both the binding and eluting buffer. The researchers are requested to share the purification profile

As suggested by the reviewer, we have made the purification protocol clearer by detailing the previously shortened steps and providing all components of buffers. Please note that, we have used Tris/NaCl-based buffers for all our purification protocols.

• "The results showed that all three crRNAs could specifically detect B. pseudomallei, but not other pathogens". Are the authors taking any range level to specify the detection limit, as it is observed form the Figure 2B: the fluorescence intensity is detected for the "S. epidermidis" and "B. thailandensis" as well apart from "B. pseudomallei"; and in Figure 2C: the fluorescence intensity is detected for the "S. pneumoniae" and "S. aureus ATCC" also on a lower scale.

The reviewer has raised a great point. This issue likely stemmed from the distorted unit on the logarithmic scale (log10), which resulted in the disproportionately elevated signals of *S. epidermidis* and *B. thailandensis* in the previous Figure 2B. We became aware that a logarithmic plot may have caused a confusion and thus replotted the graph on a linear scale (new Figure 2B-D). This will also enable readers to better differentiate the kinetics of each crRNA.

New Figure 2B-D

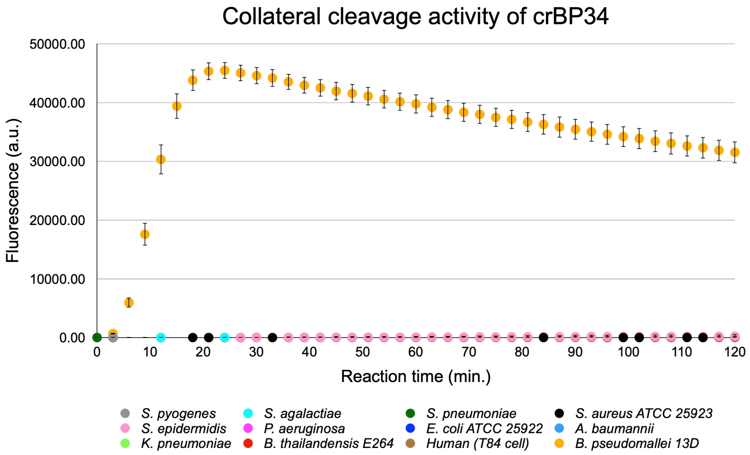

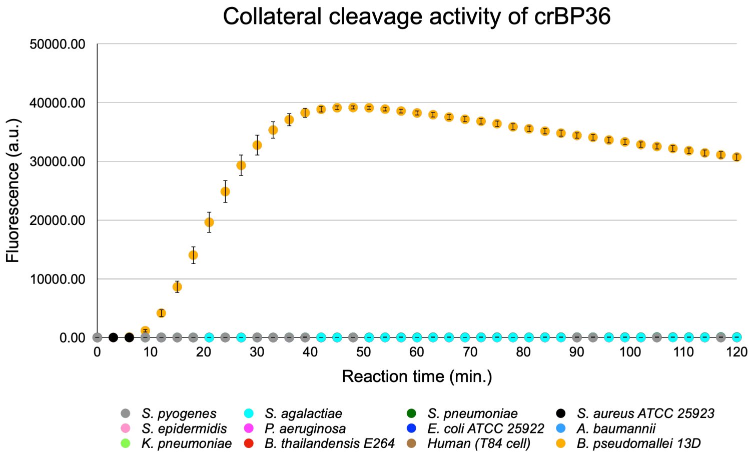

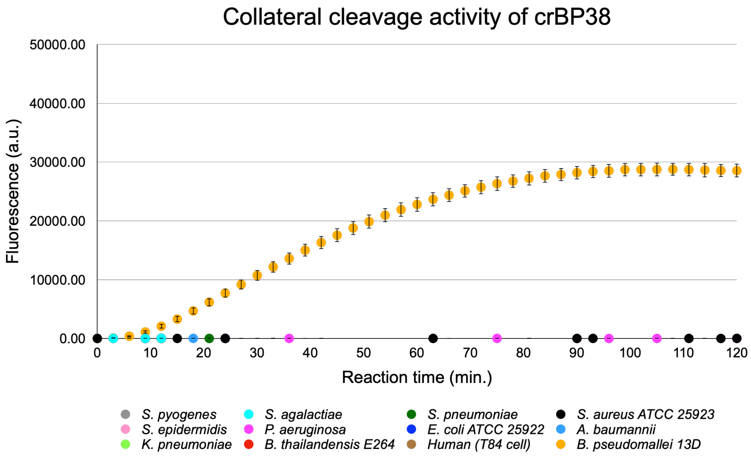

Reviewer #2: The authors had robust methods that were thoroughly explained. My concerns/questions regarding the methods are listed below.

Line 136: Define LB. Was Lennox broth or Luria Bertani broth used? Line 290 defines LB as Luria-Bertani but it would be useful to have LB defined on line 136 as well.
We have clarified that our LB was Luria Bertani, as suggested.

It was unclear to me how the authors selected crBP34, 36 and 38 from the list of candidate target sites from Fig 1D.
We thank the reviewer for this helpful comment. To clarify this, we have elaborated more on this issue (Line 411-415). The criterion was simply based on how well these target sites were amplified during RPA. We also have provided these data in Fig S1.

Fig 1 was a very helpful figure to understand the design process for the Bp specific

CRISPR RNA. I was happy to see that the authors screened the possible CRISPR sites against cross-reactive pathogens and I have two questions about this step. 1) Why did the authors not in-silico screen the final target (crBP34) against all available bacterial pathogens from NCBI. 2) If the ultimate goal of this assay is to test clinical samples why were the targets not screened against the human genome to look for interactions?
We are very pleased to learn that the reviewer found Figure 1 useful and would like to thank them for their kind encouragement.

We agree with the reviewer that a more comprehensive screening would be to include all available bacterial pathogen and human genomes from NCBI in the initial scan. However, our filtering criteria was site-specific and computationally expensive. To ensure specificity, the sequence of TTTV$N_1N_2N_3 \ldots N_{20}$ in *Bukholderia pseudomallie* must not be detected in other species with either less than four mismatches in position N_7-N_{20} or less than three mismatches in position N_1-N_6 or less than two consecutive mismatches in position N_1-N_6. This resulted in a highly stringent set of candidates at the expense of computation load and led us to limit an initial search to a smaller subset of common pathogens found in the same clinical setting as *Burkholderia pseudomallei* to ensure no cross-reaction.

In light of the reviewer's comment, we performed a post-hoc analysis to screen for the specificity of eight crRNAs targets against bacteria and human genomes. To reduce computational load, we used a WebBLAST before further screening the candidate (see item 5 in Materials and Methods) for their potential cross-reacting sites using a more relaxed threshold, namely relaxing 4th position of PAM and allowing 0-1 mismatch in N_1-N_{20} (deduced from studies in Ref 1 and 2). We found no match in any of the 40,827 archived bacterial reference genomes and in human reference genome GRCh38.p13 from NCBI. The analysis is summarized as Fig S3 and S4.

Besides, the high specificity of crRNAs, RPA primers also help add another layer of specificity to the DETECTR platform, as target sites must be able to be amplified before the CRISPR reaction.

Consistently, our data presented in Fig 2B-D showed that our detection platform with crBP34, 36 and 38 show no cross-reaction with genomic DNA from tested bacteria pathogens as well as from human (T84 cell line).

Ref 1: Kleinstiver, B. P. *et al*. Nat Biotechnol 34, 869–874 (2016)
Ref 2: Kim, D. *et al*. Nat Biotechnol 34, 863–868 (2016)

RESULTS

Reviewer #1: (same)

Reviewer #2: The results are described and presented in a clear manner. The figures are detailed and provide a nice visual of the design and validation process.
We are thankful for the reviewer's kind comment.

CONCLUSIONS

Reviewer #1: (same)

Reviewer #2: The discussion is concise and describes the power of the newly developed crBP34-DETECTR in both sensitivity and specificity. The authors also reiterated that the assay is currently validated for the detection of Bp DNA after isolation from a clinical sample and more work is needed to validate the assay on complex clinical samples.
We appreciate the reviewer's constructive comments.

EDITORIAL AND DATA PRESENTATION MODIFICATIONS

Reviewer #1: (same)
Reviewer #2: Minor:
Line 105, uncapitalize "Initial"
We have fixed this.

Line 270: uncapitalize "Primers"
We have fixed this.

Line 288: It looks like "incubate" should be in the past tense "incubated"
We have fixed this.

Line 465: "figure 2a" instead of Fig 2a
We have fixed this.

SUMMARY AND GENERAL COMMENTS

Reviewer #1: (same)

Reviewer #2: In the introduction the authors set up the issue well and why they aimed to find a cheaper, specific, fast alternative to detect Bp in clinical samples. The authors reviewed the literature and although there have been other studies that have developed a lateral flow recombinase polymerase amplification (LF-RPA) for the detection of Bp the authors of this study point out that the previously published studies did not investigate the specificity of the targets were not tested on a wider Bp population. Additionally, this study is unique from the other studies since it also uses CRISPR-Cas12a for the detection of Bp which has been validated on other pathogen species.

This study seems like a big step in the right direction for a cheaper, faster alternative for Bp detection in the clinic. The only major limitation I see with this study is the lack of validating the assay in the presence of a human DNA background. The authors speak to the limitation of the study in that clinically isolated Bp and not clinical samples (blood, urine, etc.) were not tested (lines 529-530) but they don't speak of the potential interactions that may occur with the human DNA background present in most clinical samples. With this said it should be noted that the authors do not make claims that their assay detects anything more than Bp DNA. The title is quite clear that this assay is detecting Bp genomic DNA.
We thank the reviewer for this very useful comment. In response to this, we have performed the experiment in the presence of different amounts of human genomic DNA and found that the assay was negatively impacted by human genomic DNA (Fig S2). This is a known issue for nucleic acid detection assays and is consistent with other studies that found high background of contaminant DNA impair assay's specificity and sensitivity (Ref 1,2, 3). It's noteworthy that the effect of human DNA was more pronounced in a reaction with lower copy number *B. pseudomallei* DNA than that with higher copy number.

We have also added texts to discuss this issue in the manuscript. (Line 477-481 and 578-584)

Ref 1: Handschur, M., Karlic, H., Hertel, C., Pfeilstöcker, M. & Haslberger, A. G. Comp Immunol Microbiol Infect Dis 32, 207–219 (2009).
Ref 2: Mancini, N. et al. Clin Microbiol Rev 23, 235–251 (2010).
Ref 3: Döring, G., Unertl, K. & Heininger, A. Clin Chem Lab Med 46, 909–918 (2008).

The abstract is a bit misleading since the authors say that the assay can be achieved in less than 1 hour (line 34) but fail to mention that this is after isolating Bp from a clinical sample (24-48 hours) and after a DNA extraction on that pure isolate (1-4 hours).

I think the authors need to be clearer about the input material required for the assay. For example, lines 36-37 make claims of this assay being used in “poor-resourced clinical settings” which may be true one day but that is not the case yet since the assay has not been validated on clinical samples, DNA extractions from clinical samples, or mock clinical DNA samples of Bp DNA with a human DNA background. Where lines 527-530 in the discussion are clearer about this limitation. I would like to see something more like lines 527-530 in the abstract instead of lines 36-37.
We thank the reviewer for helping us improve this manuscript. We have incorporated the reviewer’s suggestions such as removing specific assay time, clarifying that an input material is purified *B. pseudomallei* DNA, as well as mentioning limitations of the assay. These changes can be found in both the abstract (Line 29-30; 34-35; 37-38) and in the introduction (Line 132, 140-143)

ADDITIONAL CHANGES

1. We have made a correction in “2. Searching for optimal CRISPR-Cas12a target sites” under the Methods section. Candidates were in fact mapped to all possible cross-reactive CRISPR-Cas12a target sites (i.e. all sites preceded with PAM TTTN) in 1,071 genomes of non-*B. pseudomallei*, rather than to their entire genomes. And, the original PAM mismatch criterion was thus removed.

2. We have accordingly edited Fig 1C to reflect the above changes in #1.

3. We have added more detail of designing RPA primers (Line 269-274).

4. We have edited one acknowledgement to comply with a writing style of an agency (Line 601-603).

5. We made an error by adding “*S. suis*”, which was not in our analysis, in Fig 1C. Thus, we have made a correction in both Fig 1C and total number of cross-reactive genomes used (Line 189).

6. We have updated a link for GitHub.

7. We have updated *in silico* detection of CRISPR-Cas target in the Materials and Methods section. This reflects additional analysis performed in response to the reviewers’ comments.

8. We have added supporting information captions for Fig S2-S4 (Line 983-985).
